# A bibliometric analysis of literatures on uterine leiomyosarcoma in the last 20 years

**DOI:** 10.3389/fonc.2024.1343533

**Published:** 2024-02-12

**Authors:** Jinhua Huang, Yu Chen, Ziyin Li, Mimi Chen, Dingwen Huang, Peixin Zhu, Xintong Han, Yi Zheng, Xiaochun Chen, Zhiying Yu

**Affiliations:** ^1^ Department of Gynecology, Shenzhen Second People’s Hospital/The First Affiliated Hospital of Shenzhen University Health Science Center, Shenzhen, Guangdong, China; ^2^ College of Medicine, Shantou University, Shantou, Guangdong, China

**Keywords:** uterine leiomyosarcoma, bibliometric analysis, gynecologic malignance, top relavant keywords, trend topic

## Abstract

**Background:**

Uterine leiomyosarcoma(uLMS) is a rare malignant tumor with low clinical specificity and poor prognosis.There are many studies related to uLMS, however, there is still a lack of metrological analyses with generalization. This study provides a bibliometric study of uLMS.

**Methods and materials:**

We chose the Web of Science (WoS) as our main database due to its extensive interdisciplinary coverage. We specifically focused on the literature from the last 20 years to ensure relevance and practicality. By utilizing the WOS core dataset and leveraging the R package “bibliometric version 4.1.0” and Citespace, we performed a comprehensive bibliometric analysis. This allowed us to pinpoint research hotspots and create visual representations, resulting in the retrieval of 2489 pertinent articles.

**Results:**

This literature review covers 2489 articles on uterine leiomyosarcoma (uLMS) from the past 20 years. Key findings include an average annual publication rate of 8.75, with a 6.07% yearly growth rate and an average citation count of 17.22. Core+Zone 2 sources contributed 1079 articles and 207 reviews, displaying a 4.98% annual growth rate. The analysis identified top journals, influential authors, and core sources, such as the prevalence of publications from the United States and the dominance of *GYNECOLOGIC ONCOLOGY* and *HENSLEY ML*. Bradford’s Law and Lotka’s Law highlighted core sources and author productivity, respectively. Thematic mapping and factorial analysis revealed research clusters, including etiology, diagnosis, treatment advancements, and surgical approaches, with prominent themes such as gemcitabine and docetaxel. Overall, this comprehensive analysis provides insights into uLMS literature trends and influential factors.

**Conclusion:**

This thorough bibliometric analysis, in its whole, illuminates the field’s guiding principles while also revealing the subtle patterns within the uLMS literature. The knowledge gained here contributes to the current discussion in uLMS and related scientific fields and provides a solid basis for future research paths.

## Introduction

Uterine leiomyosarcoma (uLMS) is a rare malignant neoplasm, presenting with an annual incidence of approximately 0.8/100,000 cases (1). It constitutes a significant proportion, accounting for 80% of all uterine sarcomas and ranging from 3% to 7% of all uterine carcinomas (2). Despite its relatively low incidence, uLMS poses a substantial threat to women’s reproductive function, and more critically, their overall health and life expectancy (3). The early stages of uLMS often lack specific symptoms, contributing to the challenges in timely detection. In advanced stages, manifestations may include irregular vaginal bleeding, abdominal pain, the presence of an abdominal mass, or compression symptoms (3). The absence of a distinct symptom profile complicates early diagnosis. Pathologists rely on evaluating morphological features such as nuclear anisotropy, tumor necrosis, and nuclear fragmentation (3). Adding to the complexity, the etiology of uLMS remains indeterminate, and current investigations into its underlying mechanisms are limited. Despite a notable body of subject-specific literature, there is a noticeable dearth of comprehensive scholarship offering an overview of the subject.

Bibliometric analyses offer a comprehensive insight into extensive academic literature. Through the quantitative analysis of citation patterns, annual publication outputs, global distribution of publications by country and institution, author productivity, collaboration patterns, and corresponding impact factors, researchers can systematically evaluate a specific research field (4) (5),Utilizing bibliometric tools such as bibliometric (6), Vosview, Citespace, and Gephi (7, 8) for visualization analysis, scholars have conducted bibliometric studies in various domains. For instance, in assessing the impact of literature, Dejian Yu et al. applied bibliometric techniques to conduct an in-depth analysis of the international journal “Information Sciences,” focusing on its literature structure, readership, and developmental trends. They comprehensively assessed the journal’s citation and co-citation data (5).In the field of environmental ecology, Lili Zhang et al. pioneered the use of recognized bibliometric indicators to analyze global articles on Carbon Neutrality, proposing potential solutions to environmental issues caused by carbon emissions (9). Renewable energy, as a crucial component of environmental conservation, faces essential challenges in its application. A bibliometric study by Lili Zhang et al. reveals that artificial intelligence-related technologies can effectively address issues related to the integration of renewable energy with power systems, including assessing transient stability. This research contributes to a better understanding of the evolution in this field, sparking more comprehensive inspiration for further exploration (10). On the other hand, emerging contaminants represent a current focal point of research. Yixia Chen et al. conducted a thorough review of 10,605 publications on the web using bibliometric analysis. The findings indicate that existing purification and removal techniques, such as ozonation or membrane filtration, are effective in removing pharmaceutical compounds from water sources. However, the study also highlights the current challenges in efficiently detecting newly emerging pollutants and optimizing removal methods, which are crucial for guiding future environmental protection efforts (11). Within operational research, the Preference Ranking Organization Method for Enrichment Evaluations (PROMETHEE) has gained recognition for its flexibility in integrating qualitative and quantitative indicators in complex decision-making. Dejian Yu et al. employed bibliometric methods to delve into PROMETHEE, finding researchers inclined toward improving PROMETHEE within the contextual backdrop provided by practical environments (12).In addressing multi-criteria decision-making (MCDM) problems, Analytic Hierarchy Process (AHP) and Analytic Network Process (ANP) have been widely applied. Dejian Yu et al. employed bibliometric methods to analyze this field, providing valuable guidance for beginners in AHP/ANP studies to swiftly understand authoritative countries/regions and institutions (13).In recent years, bibliometrics has played a crucial role in the study of the novel coronavirus. Meihui Zhon et al. conducted a mixed qualitative and quantitative analysis of literature related to the economic aspects of the pandemic, with clustering analysis yielding conclusions aligned with the current economic situation (8). Furthermore, in the medical field, bibliometrics has been extensively used in various areas such as cancer research (14), periodontology (15), nanomedicine (16), and psychocardiology (17). In conclusion, bibliometrics has emerged as a vital tool for investigating the development of various fields (18) and serves as an essential technique for both qualitative and quantitative analyses of scientific research. Its visualization techniques enable an intuitive exploration of research profiles in related fields (19).

## Materials and methods

**Figure 1:**
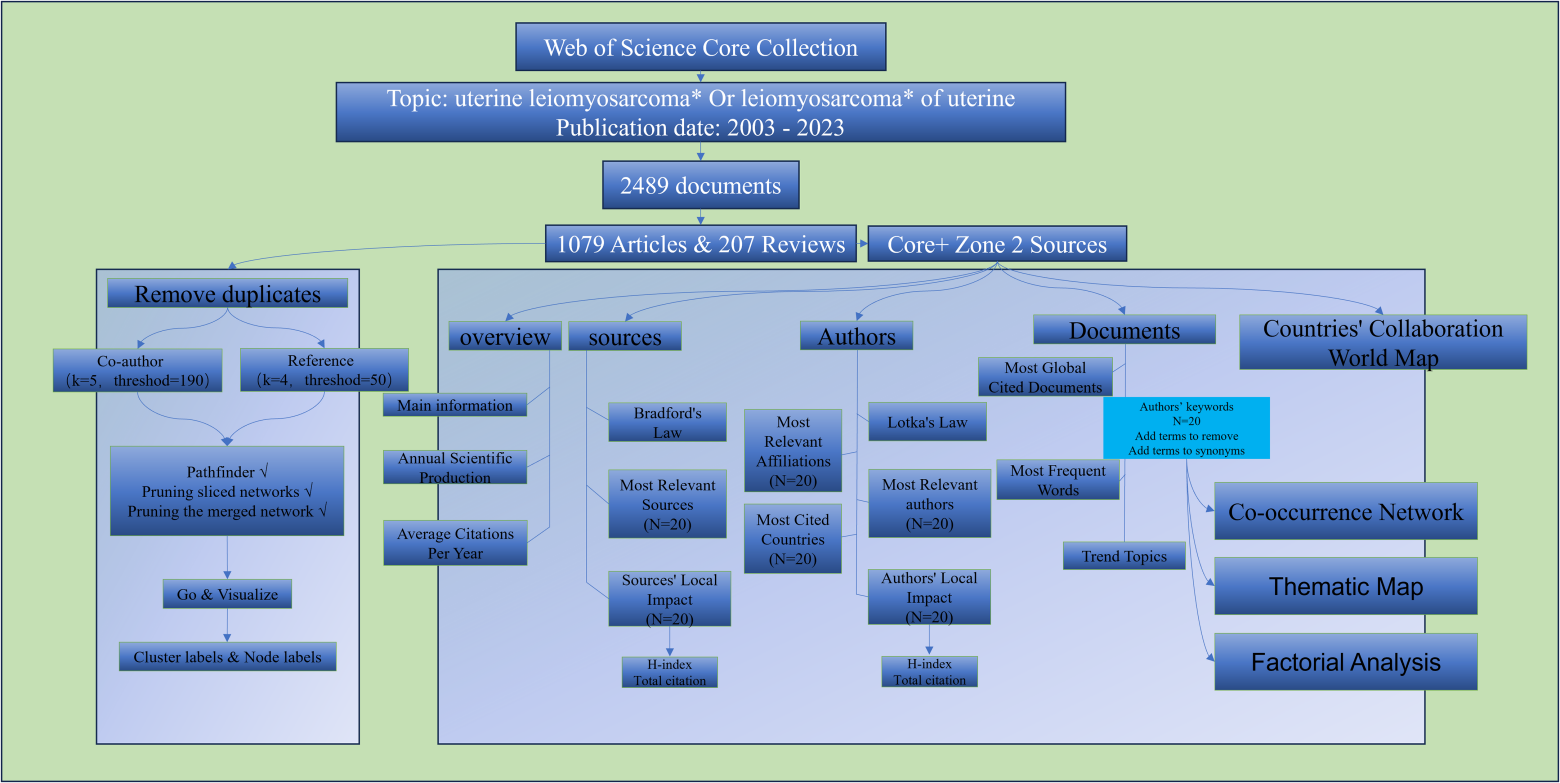
Search strategy and analysis route.

For the selection of databases, we took into consideration several globally recognized databases, including WOS, Pubmed, Medline, Scopus, among others. Among these databases, the Web of Science (WoS) served as our primary choice due to its comprehensive coverage across various disciplines, offering all-encompassing support for academic research (20). In determining the timespan for our study, we opted to focus on literature from the past 20 years. This decision aimed to ensure the timeliness of our research, allowing for an accurate reflection of the latest trends in the field. The selection of this timespan also facilitated effective control over the scale and complexity of the study, rendering it more operationally feasible. In conclusion, the search was conducted using the WOS core dataset, which spans the years 2003 to 2023 and has only English-language literature. “Uterine leiomyosarcoma* Or leiomyosarcoma* of uterine” was the search term used. After making all of these decisions, we were left with 2,490 pertinent articles. Except for articles; early access, article; proceedings paper, correction, editorial material, editorial material; early access, letter, meeting abstract and review; and early access, restricted to all languages for comprehensiveness of the data, all articles and reviews that published Core+ Zone 2 sources were included. Specific retrieval strategies are shown in **Figure 1**.

For bibliometric analysis, the creation of popular topics, and the identification of research hotspots, we employed the R package “bibliometric version 4.1.0” (6, 21). Citespace was employed to analyze co-cited authors and co-cited reference, representing two focal points of the software (22). As shown in **Figure 1**, this is our retrieval and analysis workflow, with specific parameters and steps illustrated in the figure. Figures and tables presented in this article were generated using “bibliometric version 4.1.0” and Citespace.

## Result

### Overview of literature on uLMS

We searched the Web of Science Core Collection (WoSCC) for articles published in the last 20 years that dealt with uterine leiomyosarcoma (uLMS). Of the documents found, there are 2489 documents in total. 10320 authors and 576 sources in total; single-author works made up just 0.66% of the total. 3,168 author’s keywords were utilized by authors in their articles. With an average of 8.75documents released annually and an average of 17.22 citations per document, the yearly growth rate of papers was 6.07%.

**Figure 2:**
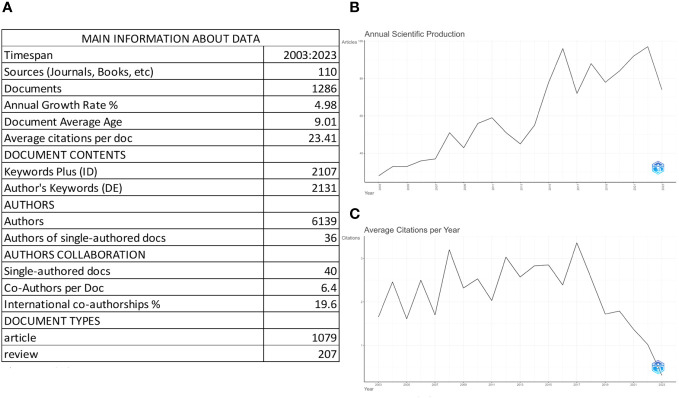
The overview of Uterine Leiomyosarcomas (uLMS) Documents. **(A)** Overview of Key Data on uLMS Documents. The table provides a comprehensive overview of documents related to uLMS retrieved from the Web of Science (WOS) spanning from 2003 to 2023. These documents are sourced from 110 journals and involve 6139 authors, contributing to a total of 1286 documents. Among these, 1079 are articles, and 207 are reviews. The dataset comprises a wealth of information, including 2107 Keyword Plus entries and 2131 Author’s Keywords. Notably, there has been a steady Annual Growth Rate of 4.98%, reflecting the evolving interest and research in uLMS over the years. On average, there are 9.01 articles related to uLMS published each year, and these publications garner an average of 23.41 citations annually. This comprehensive analysis sheds light on the dynamic landscape of uLMS research, highlighting its interdisciplinary nature and the ongoing scholarly contributions to this field. **(B)** Annual Scientific production of uLMS. There is a discernible upward trajectory in the annual publication volume of uLMS spanning from 2003 to 2023. This trend was particularly pronounced from 2013 to 2016 and has consistently maintained levels surpassing 70 articles per year since then. **(C)** Average Citaion Per Year of uLMS. From 2003 to 2023, the average annual citations for uLMS literature exhibit a stable trend.

We filtered all papers using the bibliometrics software in order to collect important data from reviews and articles that were especially relevant to uLMS from Core+Zone 2 sources. As can be seen in **Figure 2A**, 6139 authors submitted a total of 1,079 articles and 207 reviews from 110 distinct sources (books, journals, etc.). Forty of these, or 3.11% of the total, were publications with just one author.

The yearly growth rate of the articles and reviews from Core+Zone 2 Sources was 4.98%, with an average of 9.01 publications each year and an average citation count of 23.41 per document. Across all papers, 2131 keywords were utilized, matching 2107 keyword plus phrases. The trend in uLMS-related scientific papers throughout time is seen in **Figure 2B**. There has been a consistent yearly increase in the quantity of publications on uLMS, with a significant surge from 2013 to 2016. Subsequently, the annual publishing rate has continuously stayed over 72 articles. Average Citaion Per Year of uLMS is shown at **Figure 2C**. From 2003 to 2023, the average annual citations per article is basically stable at 1.67 times.

### Sources analysis

**Figure 3:**
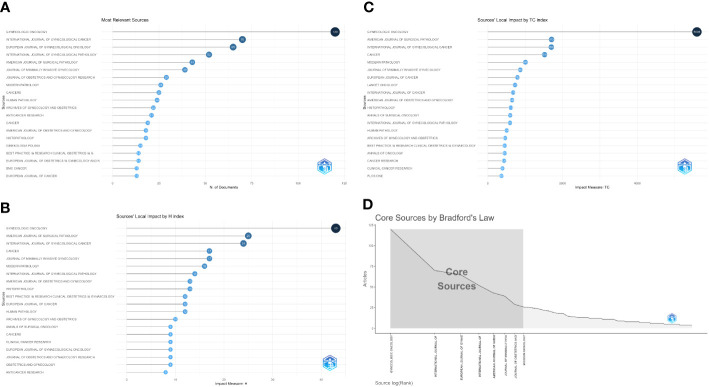
Analysis of the sources. **(A)** Top 20 sources of the most relevant sources. **(B)** Top 20 influential journals by H-index. **(C)** Top 20 journals by Total citaiton. **(D)** Core Sources by Bradford’s Law.

The top 10 journals with the highest number of publications in the field of uLMS (uterine leiomyosarcoma) are as follows: *GYNECOLOGIC ONCOLOGY*, *INTERNATIONAL JOURNAL OF GYNECOLOGICAL CANCER*, *EUROPEAN JOURNAL OF GYNAECOLOGICAL ONCOLOGY*, *INTERNATIONAL JOURNAL OF GYNECOLOGICAL PATHOLOGY*, *AMERICAN JOURNAL OF SURGICAL PATHOLOGY*, *JOURNAL OF MINIMALLY INVASIVE GYNECOLOGY*, *JOURNAL OF OBSTETRICS AND GYNAECOLOGY RESEARCH*, *MODERN PATHOLOGY*, *CANCERS*, *HUMAN PATHOLOGY*. Notably, *GYNECOLOGIC ONCOLOGY* leads with 121 articles, followed by *INTERNATIONAL JOURNAL OF GYNECOLOGICAL CANCER* and *EUROPEAN JOURNAL OF GYNAECOLOGICAL ONCOLOGY* with 71 and 65 articles, respectively, which is shown at **Figure 3A**.

Whether based on H-index or total citations (TC) as the reference standard, the top three influential journals in the field of uLMS are *GYNECOLOGIC ONCOLOGY*, *AMERICAN JOURNAL OF SURGICAL PATHOLOGY*, and *INTERNATIONAL JOURNAL OF GYNECOLOGICAL CANCER*. These journals are at the forefront of research and hold significant influence in the field, as evidenced by current research trends. **Figures 3B, C** illustrates top 20 influential journals by H-index and total citation.

According to Bradford’s Law, the core sources in the field of uLMS are as follows, as shown in **Figure 3D**: *GYNECOLOGIC ONCOLOGY*, *INTERNATIONAL JOURNAL OF GYNECOLOGICAL CANCER*, *EUROPEAN JOURNAL OF GYNAECOLOGICAL ONCOLOGY*, *INTERNATIONAL JOURNAL OF GYNECOLOGICAL PATHOLOGY*, *AMERICAN JOURNAL OF SURGICAL PATHOLOGY*, *JOURNAL OF MINIMALLY INVASIVE GYNECOLOGY*, *JOURNAL OF OBSTETRICS AND GYNAECOLOGY RESEARCH* and *MODERN PATHOLOGY*, The shaded area in **Figure 3D** represents the core sources, indicating their central importance in the field.

### Authors analysis

**Figure 4:**
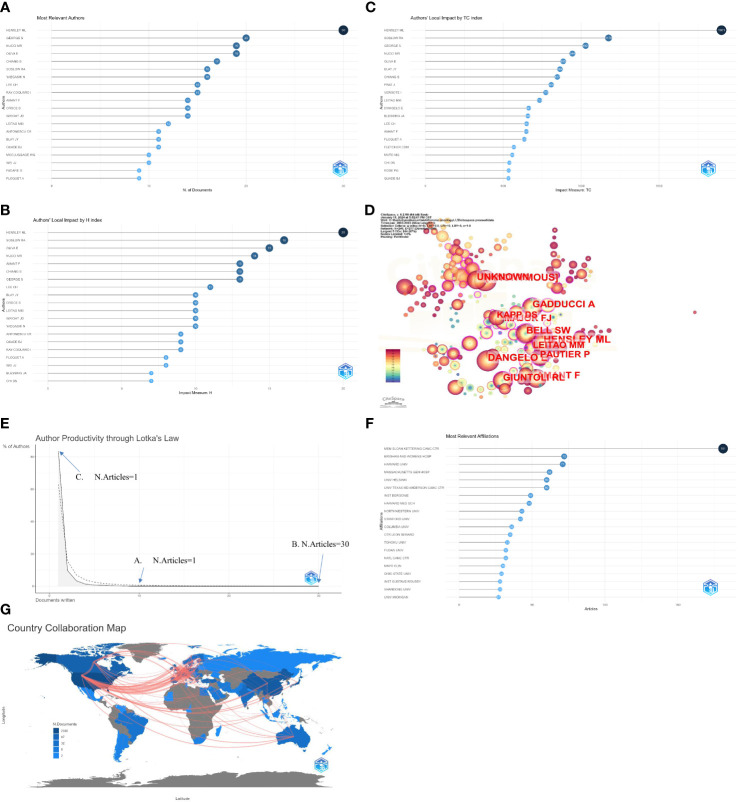
Analysis of the author. **(A)** The top 20 most published authors. **(B)** List of the top 20 most influential authors by H-index. **(C)** List of the top 20 most influential authors by Total citaiton. **(D)** Top 12 Co-authors analyzed by Citespace. **(E)** Analysis of author productivity by Lotka’s Law. In total, several authors produced at least 10 or more publications (**A**. n=18, 0.30%), one author wrote 30 (**B**. n=1, <0.01%), and the majority of the authors published only one document (**C**. n=5121, 83.44%). **(F)** List of the top 20 institution with most publishes. **(G)**. The publication output by country and their Collaboration in a world map. the top three countries in terms of the number of articles are USA (2030), Japan (622), and China (520), the frequency of country-to-country contacts is also clearly shown, with the highest frequency of cooperation being USA to Canada (33 times), USA to China (21 times), and USA to Germany (21 times).

**Figure 4A** illustrates the top 20 most published experts. The top 10 authors with the highest number of publications in the field include *HENSLEY ML*, *GEORGE S*, *NUCCI MR*, *OLIVA E*, *CHIANG S*, *SOSLOW RA*, *YAEGASHI N*, *LEE CH*, *RAY-COQUARD I* and *AMANT F*. Leading the pack is *HENSLEY ML* with a total of 30 articles, closely followed by *GEORGE S* with 20, and then OLIVA E and NUCCI MR each with 19 published articles. From H-index perspectives, the three most influential authors are *HENSLEY ML* (H-index=20), *SOSLOW RA* (H-index=16), and *OLIVA E* (H-index=15), which shows at **Figure 4B**. On the other hand, From total citation perspectives, the three most influential authors are *HENSLEY ML* (TC=1901), *SOSLOW RA* (TC=1176), and *GEORGE S* (TC=1029), which shows at **Figure 4C**. Through the use of CiteSpace analysis, it is evident that the top co-authors align closely with those identified through traditional bibliographic methods, with *HENSLEY ML* maintaining the highest ranking among authors (**Figure 4D**).

Analyzing the productivity of authors using Lotka’s Law (**Figure 4E**) reveals that while several authors have produced at least 10 or more publications (n=18, 0.30%), the majority have only published one paper (n=5121, 83.44%), with one author having written 30 articles. The institutions with the highest number of publications are displayed in (**Figure 4F**), with *MEMORIAL SLOAN KETTERING CANCER CENTER*, *BRIGHAM AND WOMEN’S HOSPITAL* and *HARVARD UNIVERSITY* ranking in the top three.

To analyze publication output by country, we combined it with a world map and found that the United States had the highest number of publications, followed by Japan and China in third place. It is noteworthy that most institutions are also from the United States. **Figure 4G** primarily describes the world map of the publication output by country and their collaboration.

### Document analysis

**Figure 5:**
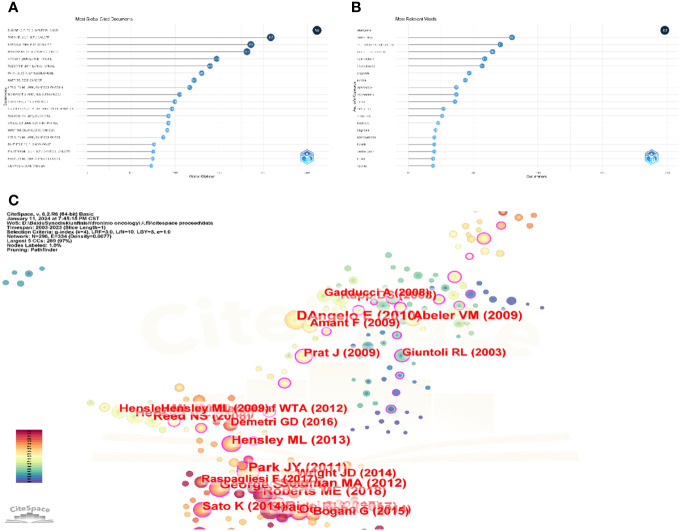
analysis of document. **(A)** Top twenty most global cited documents. These are the top 20 most cited articles in the world. The most cited article in the world is D’ANGELO E, 2010, GYNECOL ONCOL, Doi is 10.1016/j.ygyno.2009.09.023 with a total of 511 citations. **(B)** Top 20 relevant keyword of uLMS. The top 20 most frequent keywords in the uLMS field, in order of frequency, were leiomyoma(195), morcellation(82), immunohistochemistry(65), endometrial stromal sarcoma(63), hysterectomy(61), chemotherapy(58), prognosis(46), survival(44), laparoscopy(38), myomectomy(38), stump(36), recurrence(27), metastasis(26), treatment(23), fibroids(22), diagnosis(21), trabectedin(20), uterine cancer(20), fibroid(19), gemcitabine(19). **(C)** The top 13 publications with the most citations, threshold by citation = 50.

In all, 1079 papers and 207 reviews were examined. The top 20 internationally cited papers are shown in **Figure 5A**. D’ANGELO E, 2010, GYNECOL ONCOL, TORO JR, 2006, INT J CANCER, and REED NS, 2008, EUR J CANCER are the top three most cited publications. Four of these 20 articles are reviews, and the remaining 16 are original works. A review paper from the American journal Gynecologic Oncology has the highest ranking. It focuses on clinical and pathological aspects, immunohistochemical and molecular biology characteristics, prognosis, and management of uLMS. It mainly discusses the revised FIGO staging for uLMS (23). There have been 511 citations for it overall. The top 13 publications with the most citations—all of which have received more than 50 citations—can also be easily identified through Citespace analysis (**Figure 5C**).

**Figure 6:**
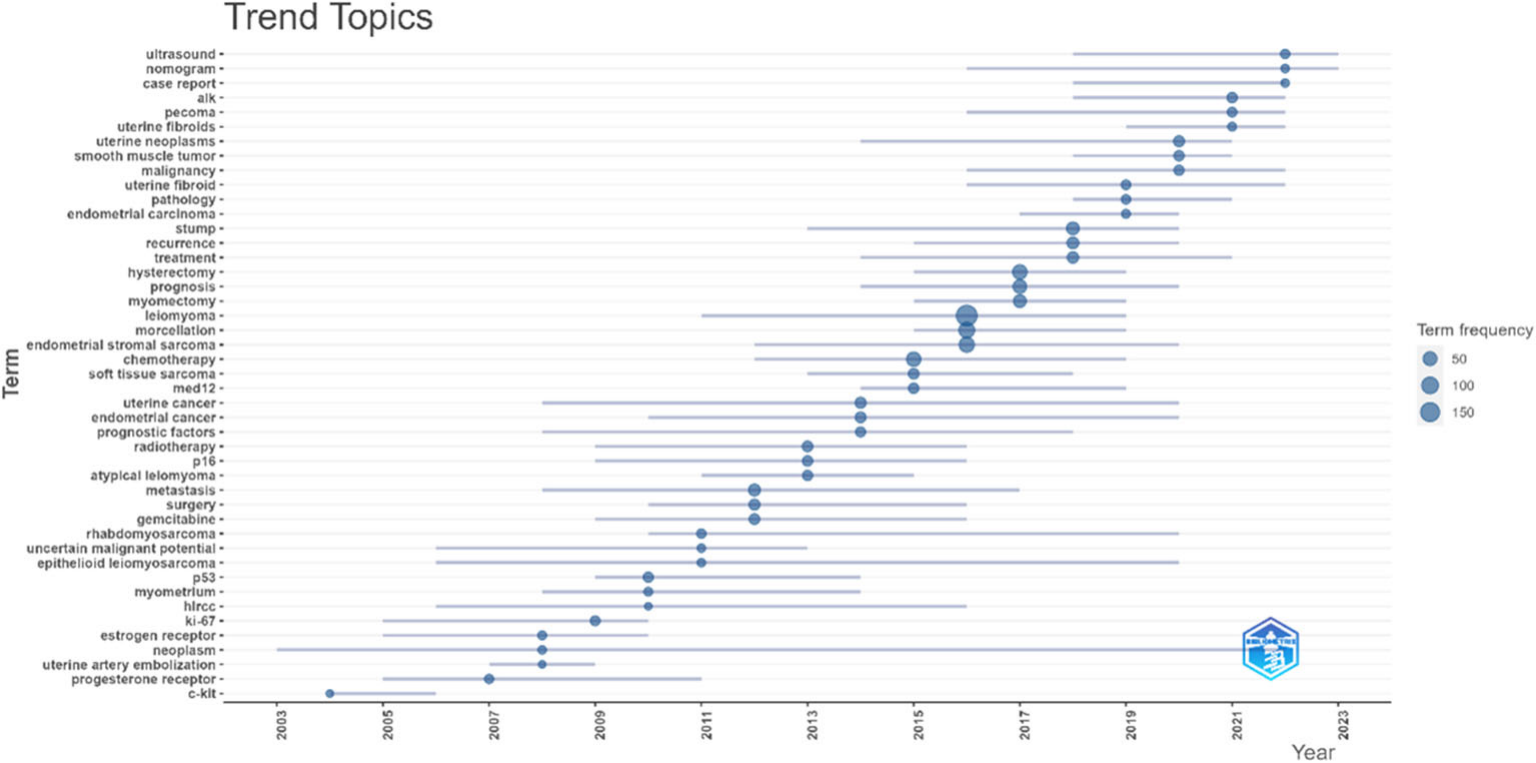
Trend Topics of uLMS in the last 20 years. This figure illustrates the trend of occurrence of the most frequently used words for uLMS over the past 20 years. The size of the circles represents the frequency of occurrence of the words, and the length of the line represents the duration of the occurrence of the words. From the figure, it can be observed that from 2021 onwards, the most popular words are ultrasound, alk, cell cycle case report and nomogram; while the duration of cell cycle and nomogram is not yet over by 2023.

Based on our acquired knowledge, we eliminated keywords associated with the retrieval approach, including uterine, uterus, uterine leiomyosarcoma, and other nominal terms that lacked importance for our investigation. Certain assimilation procedures were carried out concurrently. For instance, leiomyomas and leiomyoma were interchangeable. The 20 terms with the highest frequency were identified, and leiomyomas was the most frequently occurring (198 times), followed by morcellation. endometrial stromal sarcoma, myomectomy, immunohistochemistry, prognosis, survival, hysterectomy, chemotherapy, etc., as illustrated in **Figure 5B**. The shifting pattern of popular keywords associated with uterine leiomyosarcoma over the last 20 years is shown in **Figure 6**. It can be seen that the research hotspots of uLMS in recent 3 years are roughly nomogram, case report, cell cycle, histoathology, uterine fibroid, inflammatory myofibroblastic tumor, alk and ultrasound.

From the publications, we chose 197 case reports and 55 clinical studies using Excel’s filtering feature. The remaining papers were divided into basic research and other categories because they could not be properly classified. There were seven phase III clinical trials and thirty-four phase II clinical trials among the clinical trials, the majority of which focused on chemotherapy. With a skewed distribution, a median of 42, a mean of 71.62, and a standard deviation of 94.00, the sample sizes varied from 9 to 577. Out of all clinical trials, only eight (six phase III and three phase II) had a sample size greater than 100 patients.

### Conceptual structure

**Figure 7:**
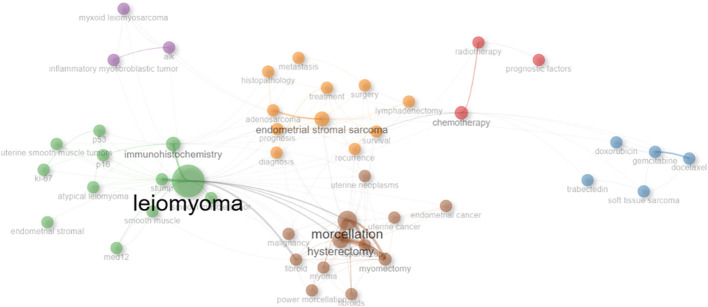
Co-occurrence network of keywords of uLMS. Key descriptive terms and search-related phrases, including prognosis, survival, management, experience, metastasis, surgery, immunohistochemistry, and recurrence, are included in Cluster 1(yellow area). Cluster 2 (brown area) is devoted to surgical techniques, specifically laparoscopy, mastectomy, and hysterectomy. Atypical leiomyomas, immunohistochemistry and related molecular markers like med12, alk, ki-67, p16 and p53 are the main subjects of Cluster 3 and 4(green and purple area). Gemcitabine and docetaxel are prominent in Clusters 5 and 6 (blue and red areas), which are linked to radiotherapy and chemotherapy, including numerous regimens of chemotherapy.

A co-occurrence network of keywords detected in uLMS-related publications is shown in **Figure 7**. The strength of the relationship between two nodes is shown by their distance from one another, and the size of each node shows the frequency of the keyword. The same color signifies a cluster of keywords that are closer together. Key descriptive terms and search-related phrases, including prognosis, survival, management, experience, metastasis, surgery, immunohistochemistry, and recurrence, are included in Cluster 1(yellow area). Cluster 2 (brown area) is devoted to surgical techniques, specifically laparoscopy, mastectomy, and hysterectomy. Atypical leiomyomas, immunohistochemistry and related molecular markers like med12, alk, ki-67, p16 and p53 are the main subjects of Cluster 3 and 4(green and purple area). Gemcitabine and docetaxel are prominent in Clusters 5 and 6 (blue and red areas), which are linked to radiotherapy and chemotherapy, including numerous regimens of chemotherapy.

**Figure 8:**
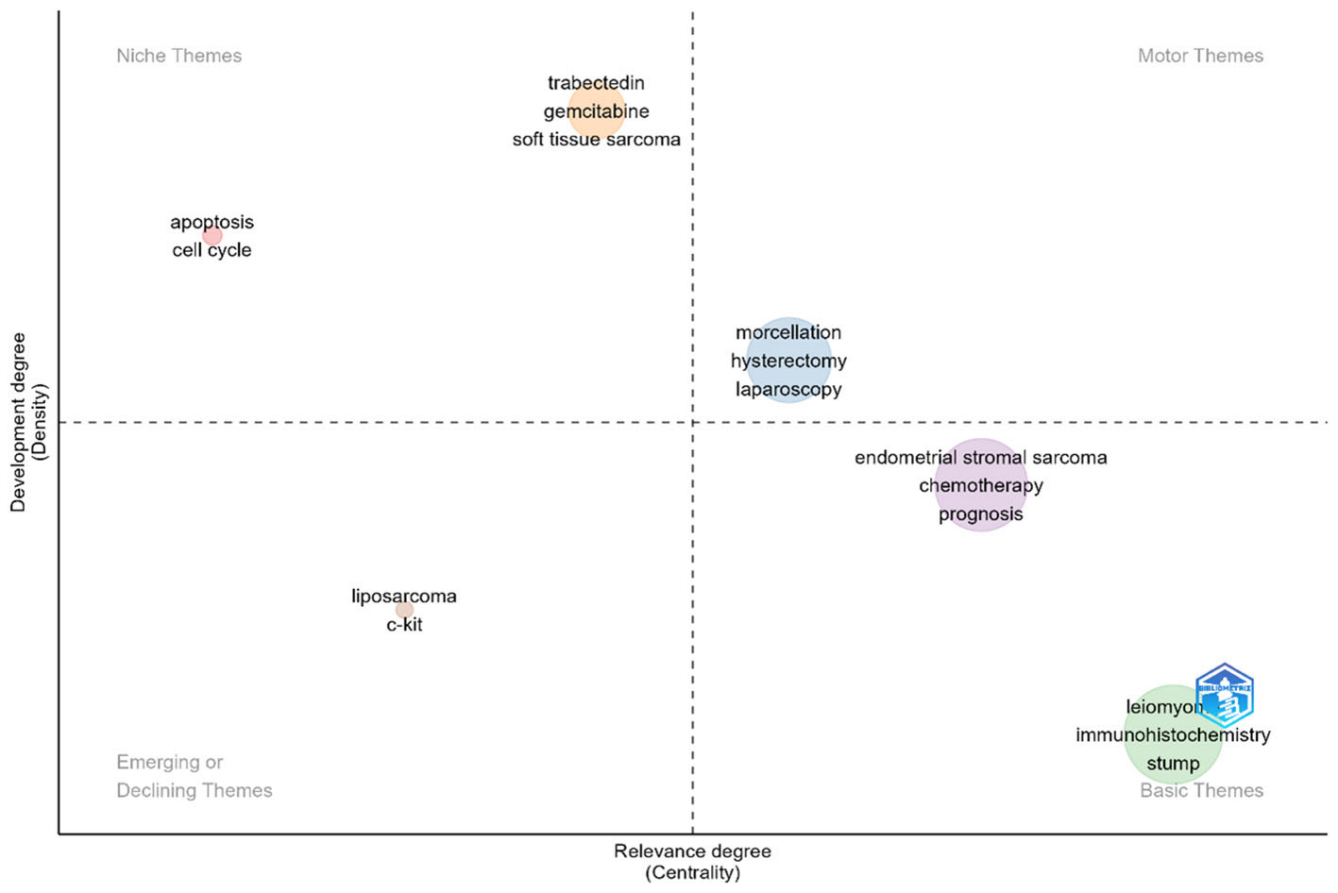
Thematic Map of keywords of uLMS. The X-axis represents centrality, while Y-axis represents density. The first quadrant inclde morcellation, hysterectomy, and laparoscopy. The second quadrant include apoptosis, cell cycle, trabectedin, gemcitabine, etc,. The third quadrant include liposarcoma and c-kit. The fourth quadrant include endometrial stromal sarcoma, chemotherapy, prognosis, and leiomyoma, immunohistochemistry and stump.

A thematic map analyzed by Bibliometric Backage is **Figure 8**. The X-axis represents centrality, indicating the level of interaction between network clusters compared to other clusters, and provides information about the importance of the themes, while Y-axis represents density, which measures the internal strength of a clustered network and can be considered as a measure of theme development. As a result, the motor-related themes, which are developed and significant themes in the research field, are identified in the first quadrant (Motor Themes). This quadrant contains terms like laparoscopy, hysterectomy, and morcellation. Plotting highly developed and isolated themes that are not highly significant in the field is done in the second quadrant (Niche Themes). This quadrant contains terms like apoptosis, cell cycle, trabectedin, gemcitabine, etc. Themes that are on the periphery and have poor development are seen in the third quadrant, titled “Emerging or Declining Themes.” This sector contains terms like c-kit and liposarcoma. Basic and cross-cutting themes, or generic subjects spanning various field research areas, are represented by the fourth quadrant (Basic Themes). This sector contains terms like endometrial stromal sarcoma, chemotherapy, prognosis, and leiomyoma, immunohistochemistry and stump (24).

### Factoral analysis

**Figure 9:**
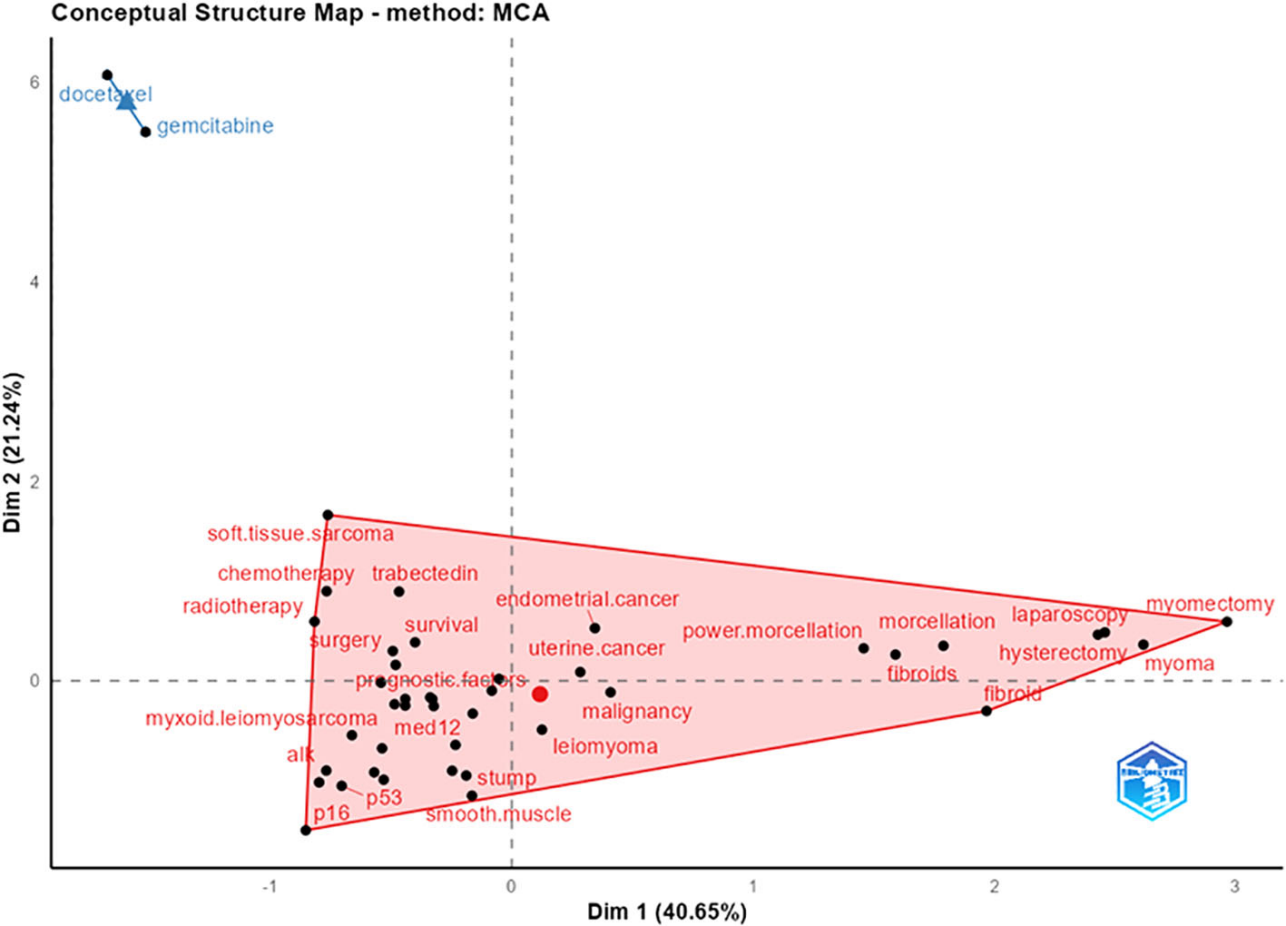
Multiple Correspondence Analysis of High-Frequency Keywords in Research Articles on uLMS. It can be observed that cluster 1 mainly focuses on four research directions: ①etiology, including the relationship between uLMS and uterine leiomyoma, as well as genetic and protein-level pathological mechanisms involving p53, p16, ALL, MED12, etc., ②diagnosis and outcome, including prognostic factors and survival rates, ③clinical research advances in the treatment of uLMS with a focus on various chemotherapy regimens, and ④surgical methods for uLMS such as laparoscopy, mastectomy, and morcellation. Cluster 2 includes two chemotherapy drugs: docetaxel and gemcitabine, two of the most important chemotherapy of uLMS.

The **Figure 9** in causal analysis combines and condenses terms from earlier research to give a multiple correspondence analysis of high-frequency keywords connected to uLMS publications. This analysis identifies two main clusters. Cluster 1 is primarily concerned with four research directions: ①etiology, which includes the connection between uterine leiomyoma and uLMS, as well as genetic and protein-level pathological mechanisms involving p53, p16, ALL, MED12, etc.; ②diagnosis and outcome, which includes prognostic factors and survival rates; ③clinical research advancements in the treatment of uLMS, with an emphasis on different chemotherapy regimens; and ④surgical approaches for uLMS, including laparoscopy, mastectomy, and morcellation. The two most significant chemotherapeutic medications for uLMS are included in Cluster 2: gemcitabine and docetaxel.

## Discussion

To the best of our knowledge, this work is the only bibliometric analysis of uterine leiomyosarcoma that is currently available. First off, our analysis was extremely thorough, covering 2489 items from the Web of Science Core Collection, comprising a variety of research formats, including articles and reviews. Our study’s scope was greater than that of systematic reviews and meta-analyses, which allowed for a more thorough investigation with less room for subjectivity and researcher bias. In terms of objectivity, the bibliometric study used a hybrid qualitative and quantitative research methodology, extracting key indicators such as total citations, H-index, and overall publishing output using appropriate software. This method improved our results’ neutrality and allowed for a more precise evaluation of the state of the field’s research on uterine leiomyosarcoma. Finally, in terms of findings, we were able to assess current research trends thanks to our application of word trend analysis. Our findings are more objective than those of other studies and demonstrate the level of attention paid to research hotspots using visual aids. Data on scientific achievements in the field of uLMS indicates that research on this topic has been ongoing for the past 20 years, with an average of 61.2 publications per year and a median of 55.5 publications. The increasing volume of publications over the years suggests that research on uLMS is becoming more widespread.

**Figure 10:**
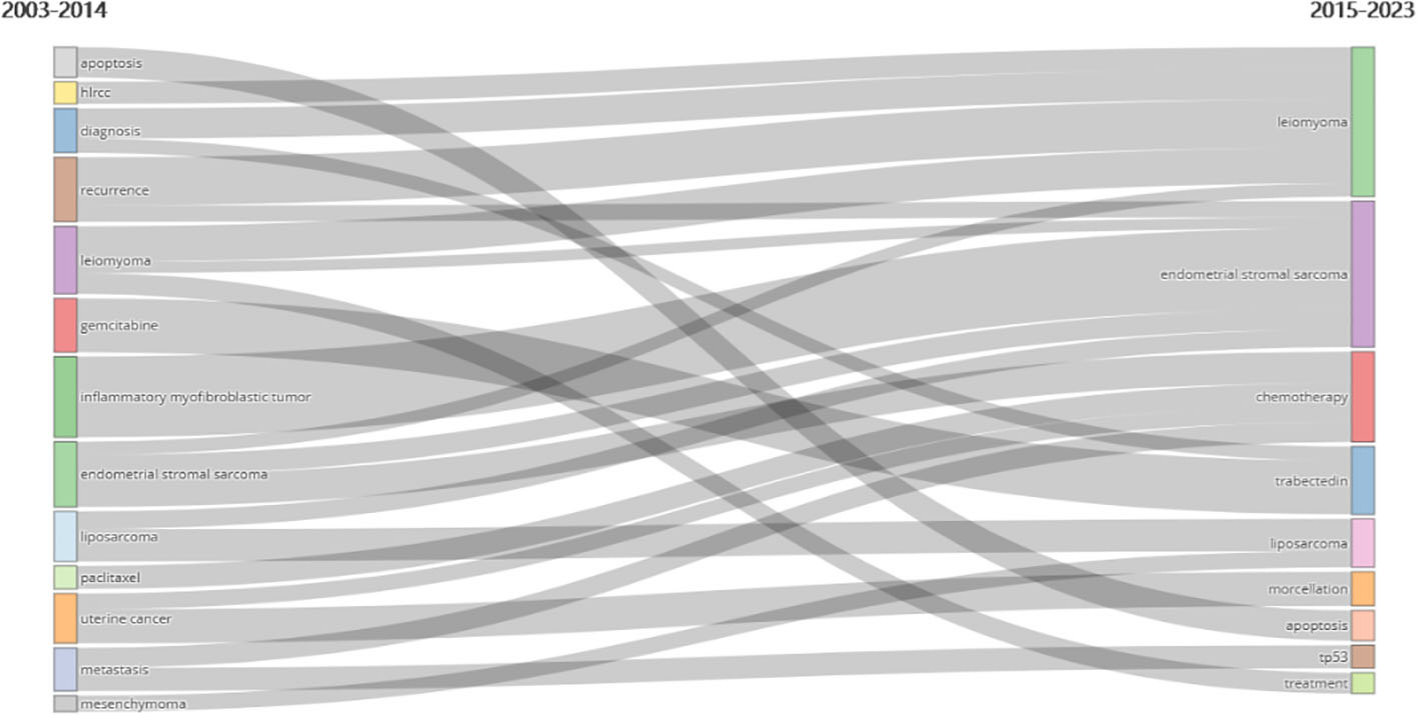
Keywords trends changes with 2015 as a time point. In the uLMS field, the key themes have shifted from apoptosis, hlrcc, diagnosis, recurrence, leiomyoma, gemcitabine, inflammatory myofibroblastic tumor, endometrical stromal sarcoma, liposarcoma, paclitaxel, uterine cancer metastasis and mesenchymoma between 2003-2014 into leiomyoma, endometrical stromal sarcoma, chemotherapy, trabectedin, liposarcoma, morcellation, apoptosis, tp53, and treatment between 2015-2023.

The graph demonstrates that, since 2015, the annual total of scientific accomplishments has continuously surpassed 72 articles. We therefore categorize the last 20 years into an early and a recent period and regard 2015 as a tipping moment. An analysis shows that 528 publications, or an average of 44 publications annually, were produced between 2003 and 2014. A total of 750 publications, or an average of 83.3 publications annually, have been published since 2015. When the Thematic Evolution is integrated, we discover that authors have demonstrated a stronger concentration on endometrial stromal sarcoma, leiomyoma, and chemotherapy (including particular chemotherapy medications like trisected) since 2015 (**Figure 10**). It is apparent from looking through some of the literature from that era that publications at that time mostly dealt with the use of morcellation in the surgical treatment of benign uterine leiomyoma. Thus, the increased usage of morcellation in minimally invasive laparoscopic surgery for benign uterine leiomyoma may be the reason for the rise in articles in this subject after 2015 (25–33).

The authors, sources, and research keywords are the main subjects of this study. With the highest TC and H-index, *HENSLEY ML* is the most prolific author in terms of publishing volume. According to Lotka’s Law, which states that the number of authors publishing n papers is 1/n^2 of those publishing one paper (34), scientific productivity may be objectively measured. A minority of core writers (n = 18, 0.30%) have published ten or more papers, according to our analysis of Lotka’s Law, but the bulk of authors (n = 5066, 83.58%) have only published one paper. According to Lotka’s Law (35), the number of writers who have made the biggest contributions to the research diminishes as the number of reports rises dramatically. Through the analysis of Lotka’s Law, we identified a small number of core authors who have published at least 10 or more publications (n=18, 0.30%), while the majority of authors have only published one paper (n=5066, 83.58%). As the number of reports significantly increases, the number of authors contributing the most to the research decreases, aligning with the predictions of Lotka’s Law (35). The top three authors with the highest H-index are *HENSLEY ML* (H-index=20), OLIVA E (H-index=15) and *SOSLOW RA* (H-index=16),. Upon analyzing the appropriate literature, we identified that *HENSLEY ML*’s uLMS research mostly focuses on treating the disease, with a focus on phase II clinical trials that comprise the majority of uLMS clinical studies. Gemcitabine plus docetaxel is one of the most used chemotherapy regimens; it can be used as a first- or second-line treatment for cancer. Current clinical studies have not demonstrated the suitability of other regimens, such as trisected, reducing plus strafing, aromas inhibitors, or Alistair, for treating uLMS or recurring uLMS. It is essential to keep in mind a number of these clinical trials share traits, including small numbers of participants and results that lack statistical confidence. Additionally, there is a shortage of relevant phase III clinical trials (36). *HENSLEY ML* has also put up novel therapeutic ideas for uLMS, such as PARP inhibitors, and has lately tried to detect the condition at the genetic level. According to a study of pertinent literature, *SOSLOW RA*, who is associated with *HENSLEY ML* (9/17), focuses his research on the diagnosis and prognosis of uLMS. He observed that the immunohistochemical manifestations of PR and ER are crucial when separating benign uterine leiomyomas from uLMS, in addition to tumor cell necrosis (TCN), nuclear anisotropy and mitotic activity should also be examined. When separating benign from malignant tumors, quantitative magnetic resonance imaging can be useful. Apart from tumor staging, which was found to be unquestionably correlated with prognosis, other results still require additional research with bigger sample sizes. The prognosis of alms is related to tumor stage, PR and AR, p53, and Bcl-2. It is noteworthy that *SOSLOW RA* has also found in recent years that PLAG1 rearrangement detection may help with uLMS diagnosis by fluorescence *in situ* hybridization, immunohistochemistry, and genomics. Furthermore, prognostic factors include the high-grade morphology and improved prognosis of a subset of endometrial mesenchymal sarcomas fused to the ZC3H7B-BCOR, as opposed to low-grade endometrial mesenchymal sarcomas (37). Through an H-index about 3, OLIVA E is the third-ranked author whose work focuses on uLMS diagnosis and differential diagnosis. The differential diagnosis of uLMS with other uncommon illnesses or cancers, such as benign uterine leiomyoma, endometrial mesenchymal tumors, uterine liposarcoma, and other tumors, has been particularly investigated by OLIVA E. He has distinguished between these conditions using methods including non-staining, *in situ* hybridization, and immunohistochemistry. Furthermore, he has studied histone deacetylase 8 (HDAC8), BRCA1, YWHAE-FAME22 rearrangement, Cyclin D1, tumor cell necrosis (TCN), and BCRO as diagnostic markers for uLMS. However, because to the very small sample size, no diagnostic marker has been proven to be more effective for uLMS or uterine sarcoma.

The journal with the highest publishing volume, H-index, and TC is *GYNECOLOGIC ONCOLOGY*, demonstrating its eminent position in the medical area. Bradford’s Law, which explains the citation distribution for a specific topic or field, can be used to identify journals with the highest citation rates in a particular field or topic (38). Our analysis revealed that the core sources for alms include *GYNECOLOGIC ONCOLOGY*, *INTERNATIONAL JOURNAL OF GYNECOLOGICAL CANCER*, *EUROPEAN JOURNAL OF GYNECOLOGICAL ONCOLOGY*, *INTERNATIONAL JOURNAL OF GYNECOLOGICAL PATHOLOGY*, *AMERICAN JOURNAL OF SURGICAL PATHOLOGY*, and *JOURNAL OF MINIMALLY INVASIVE GYNECOLOGY*. Researchers may find this information useful when choosing journals. The United States boasts a high publication and citation rate, which is consistent with the majority of the nation’s of authors and institutions being American. Additionally, the Countries’ Collaboration World Map analysis showed that there is a tendency toward the globalization of high-quality research in the field of alms, as the United States is connected to many other countries.

Keywords are essential in summarizing the main points of an document. High-frequency keywords are commonly used to identify popular topics in a specific research field (39). The high-frequency keywords for alms include leiomyomas, morcellation, endometrial stromal sarcoma, immunohistochemistry, hysterectomy, chemotherapy, prognosis, survival, laparoscopy, myomectomy. For uLMS researchers, uterine leiomyoma is unquestionably an important field of study. Uterine leiomyoma, the most common benign tumor of the uterus, seldom develops into malignancies. Based on clinical and imaging symptoms, it might be difficult to distinguish between uterine leiomyoma and the extremely malignant uLMS that affects the uterus, even though the majority of uterine leiomyomas are malignant. uLMS is typically mistakenly diagnosed as a result of pathological analysis of excised tissues, such as the uterus or benign uterine diseases like uterine leiomyoma (40). While the average age (45 years and older) at which uterine leiomyoma and uLMS occur is typically higher than that of leiomyoma(45 years and younger), both conditions can present with comparable clinical symptoms, such as heavy menstrual flow, pelvic pain, or infertility (41). The distinction between benign and malignant uterine leiomyosarcomas is typically challenging (42). Regarding imaging, ultrasonography is the most straightforward and affordable imaging test; this is related to another popular issue in our research on uterine leiomyosarcoma. When compared to transabdominal ultrasound, vaginal ultrasound provides superior views of the uterus and the morphology of uterine tumors, as well as a better evaluation of the retrograde uterus. And for easier imaging in cases of obesity, flatulence, or insufficient bladder filling, transvaginal ultrasound is generally recommended since it can better screen individuals with retroversion of the uterus, insufficient bladder expansion, excessive volumes of intestinal gas, or obesity (42). Transabdominal ultrasound is superior in evaluating large and basal leiomyomas (43), and it is worth mentioning that ultrasound has the most negative consequences for misdiagnosis of leiomyosarcoma (43); CT has limited ability to distinguish the tissue type of uterine tumor, but it can identify the pathological type of calcification, which is a condition unique to uterine leiomyoma. The focal range of uterine leiomyosarcoma and the stage of beneficial diseases can be recognized through the use of a CT plain scan, which is helpful in the interim. For a thorough assessment of uterine fibroids and for characterizing the local spread of cancerous illness, magnetic resonance imaging (MRI) is still the preferred imaging modality (44). Typical uterine leiomyomas emerge on T1 and T2-weighted MRI images as separate masses with various sizes that can be solitary or multifocal. They additionally display minimal signal intensity. Uterine leiomyosarcoma typically appears on MRI as discrete, irregular, and poorly defined masses. A low prognostic is linked to lms ≥10 cm. It varies in T1-weighted imaging and can show low or moderate signal strength; however, excessive signal strength on T1-weighted images typically indicates necrosis or bleeding, which helps identify malignant tumors. T2-weighted images with lower ADC values ranging from 0.79 ± 0.21 to 1.17 ± 0.15 (45) show medium-high signals. The LMS exhibits early and inconsistent enhancement following the intravenous injection of gadolinium-based contrast agents, frequently displaying unenhanced central necrotic regions (46). Smooth muscle content ratio is associated with the differential presentation of uterine tumors on T1T2 (47); on the other hand, the presence of other invasive features on MRI can cause misinterpretation of T1-weighted images and make it harder to distinguish between cystic and myxoid degenerative conditions on T2-weighted images (42). Imaging may have been utilized in an attempt to more accurately diagnose uterine leiomyoma and uterine leiomyosarcoma before to surgery in recent years, nevertheless the results do not appear to be particularly good, and pathology and surgery continue to be needed to confirm the final diagnosis. Pathology-wise, uterine leiomyoma can take many different forms, one of which is hyaline degeneration. This pathological type is readily misinterpreted with coagulated tumor cell necrosis in leiomyosarcoma. Furthermore, it’s critical to differentiate between cellular and associative leiomyomas, which can also be mistakenly identified as sarcomas (48); Numerous investigations have demonstrated the significance of coagulation necrosis, cytological atypia, and mitotic counts as diagnostic markers for uterine leiomyosarcoma (3). For treatment, surgery has historically been the most common option for treating uterine leiomyomas (49) and typically entails a myomectomy or hysterectomy. additionally one of the treatments for uterine leiomyoma is the release of levonorgestrel and GnRHa from the intrauterine system (50). Nonetheless, in the case of leiomyosarcoma of the uterus, a bilateral ovariectomy is necessary for women who are perimenopausal or postmenopausal, a total hysterectomy with bilateral salpingectomy is carried out in lieu of a sectional resection, and complete resection is undertaken when it is feasible. Treatment options for local recurrence consist of radiation therapy and/or modified surgery. The most popular systemic treatment is chemotherapy. When resectable, surgical treatment is still a crucial choice in the case of metastatic ailments. Local treatment of metastases should be taken into consideration when there is little to no metastatic illness. Chemotherapy is recommended in cases of stage IV and is based on a first-line treatment based on etocyclamycin. Specialized supportive care is advised for management when the patient’s overall health deteriorates significantly. For symptom relief, external palliative radiation therapy might be suggested (51). In conclusion, uterine leiomyoma has become a focal point for uterine smooth muscle tumor research. It is difficult to differentiate from uterine leiomyosarcoma in a variety of manners, including as pathological traits, imaging findings, and clinical signs. Furthermore, it is unthinkable what would happen if uterine leiomyosarcoma was mistakenly diagnosed as uterine leiomyoma. As a result, scientists keep working on this project.

The terms “morcellation,” “hysterectomy,” and “myomectomy” have a strong connection with uterine leiomyosarcoma surgical treatment. As previously stated, surgical intervention is required irrespective of the stage of uterine leiomyosarcoma. The surgical management of uterine leiomyosarcoma necessitates total excision. However, diagnosing uterine leiomyosarcoma prior to surgery is extremely difficult. After surgical excision, the majority of cases are diagnosed pathologically; prior to before that, patients may be diagnosed with benign conditions like leiomyoma or uterine adenomyosis. Guidelines call for myomectomy and hysterectomy. Because tumors that are harmless and benign, most individuals decline to have a hysterectomy. If postoperative pathology confirms leiomyosarcoma of the uterus, however, remedial surgery is frequently required (51). According to recent cross-sectional research, the disease-free survival (DFS) rates for uterine leiomyosarcoma following surgical treatment are 46% and 55%, respectively, after five and ten years. After five and 10 years, the overall survival rates were 34% and 47%, respectively. Complete resection was the most significant factor linked to distant recurrence (hazard ratio [HR] 1.91; 95% confidence interval [CI] 1.22-2.97); inadequate resection was the most significant factor linked to global recurrence (hazard ratio [HR] 2.87; 95% CI 1.91-4.31). Tumor residue following any treatment (HR 4.59; 95% CI 2.51-8.40), partial resection (HR 3.68; 95% CI 2.44-5.56), involvement of the tumor margin (HR 2.41; 95% CI 1.64-3.55), and adjuvant chemotherapy (HR 1.91; 95% CI 1.31-2.78) were the factors most closely linked to overall survival (52). This demonstrates the significance of total tumor excision in cases with uterine leiomyosarcoma. Laparoscopic surgery is now one of the most common surgical procedures for these benign diseases due to the ongoing advancements in minimally invasive technology. Numerous minimally invasive devices have also been developed recently, with morcellation playing a significant role in several of them. Using a surgical technique called morcellation, fibroids or the uterus are cut into tiny pieces. With the goal to remove the tissue utilizing laparoscopic instruments or tiny incisions. Nonetheless, there is a chance that the tumor will spread during the comminution process (53). This is becoming increasingly concerning when surgeons treat occult uterine sarcomas by mistakenly performing uterine division and myomectomy (54). Because morcellation has the potential to promote the spread of potentially malignant fragments, a safety communication from the U.S. Food and Drug Administration (FDA) in 2014 warned against using morcellation after minimally invasive surgery (55), for any uterine malignancy, and the probability of fractionated uterine malignancy is 1 in 352. The probability of developing uterine cancer was 1 in 498 for uterine leiomyosarcoma (56). To summarize, the author feels that scientists should start with the preoperative imaging examination and work to increase the likelihood of a clear diagnosis as much as possible before surgery. This will help to determine the surgical plan to the greatest extent possible and will also help to reduce recurrence, which is especially important.

One technique for identifying and measuring particular proteins in cells or tissues is immunohistochemistry. Medical professionals and scientists can learn more about the pathophysiology of linked disorders  as well as the functionality of particular proteins in cells and tissues by using immunohistochemical analysis, which is widely utilized in biomedical research and clinical diagnostics (57). Immunohistochemical analysis was used to assess the B-cell lymphoma 2 (Bcl-2) protein, and it was found that while its absence was not a diagnostic signal for uLMS, it was a significant prognostic factor (58). Immunohistochemical research also indicated that STMN1, MKI67 (59) and Androgen Receptor (60) are possible indicators for the diagnosis of uterine leiomyosarcoma. In order to investigate the involvement of tumor endothelial marker 1 (TEM1) and matrix metalloproteinase 2 (MMP-2) in the development of uterine sarcoma, immunohistochemistry analysis is also used. Research has revealed a beneficial link between the co-expression of TEM1 and MMP-2 in uterine leiomyosarcoma specimens. Additionally, by influencing MMP-2 production and extracellular matrix (ECM) remodeling, TEM1 accelerates the development of uterine sarcomas (61). The study that discovered that overexpression of Cyclin-dependent kinase subunit 2 encouraged the tumor growth of uterine leiomyosarcoma and predicted a poor prognosis also involved immunohistochemical analysis in a similar way. Additionally, inflammatory myofibroblastic tumors and uLMS have been distinguished from one another using immunohistochemical detection of aberrant P53 and P16 (62). Currently, there seems to be paucity of diagnostic immunohistochemistry analysis and the shape of uterine leiomyosarcoma is still difficult to understand. Researchers are still working on this, evaluating the genome-wide distribution of uterine LMS with targeted next-generation sequencing in order to investigate the consistency of the results of LMS immunohistochemical examination. TP53, RB1, ATRX, PTEN, CDKN2A, or MDM2 were the subjects of ≥1 chromosomal mutation in 94% of LMS, and ≥2 modifications in 80% of these cases. The test cohort is divided into initial p53, Rb, PTEN, and ATRX groups, followed by abnormality-free DAXX, MTAP, and MDM2 groups. In 75%, 88%, 44%, and 38% of LMS, respectively, abnormal expression of p53, Rb, PTEN, and ATRX IHC was discovered. IHC values for two or more of these markers were abnormal in eighty-one percent of LMS patients. There was just one IHC abnormality involving these markers in STUMP. In leiomyoma, there were no immunohistochemistry anomalies. Pathologists’ interpretation of immunohistochemistry results (κ = 0.97) and the correlation between immunohistochemical results and NGS results (κ = 0.941) was well agreed upon (63). Researchers investigating the potential therapeutic benefits of uterine leiomyosarcoma have discovered that a combination of Pazopanib and heat inhibits the production of histone acetyltransferase 1, which in turn inhibits the growth of uterine leiomyosarcoma (HAT1). In comparison with leiomyoma, leiomyosarcoma had greater levels of HAT1 expression. Poor clinical outcomes are linked to elevated HAT1 expression. Immunohistochemical analysis is a vital tool for assessing HAT1 expression in research (64). It can be observed that immunohistochemical analysis plays a significant role in clarifying the diagnosis of uterine leiomyosarcoma and evaluating its prognosis. The problem with current research, however, is that while some possible biomarkers have arisen, there are currently no adequate biomarkers for diagnosis and prognosis because of the disease’s rarity and heterogeneity (63). In addition, miRNA have been shown in recent years to demonstrate differential expression in uterine sarcoma cell lines and interact with particular genes, which are related with tumorigenesis and cancer progression (65). Additional methods need to be used because immunohistochemical analysis appears to be ineffective when it comes to mirnas. In order to better understand the relevant mechanism of uterine leiomyosarcoma and identify potential molecular markers that could aid in clinical diagnosis, follow-up targeted therapy, and prognostic improvement, the author believes that future research on uterine leiomyosarcoma should concentrate on the use of gene, RNA, and related technologies like immunohistochemical analysis. Few research are prospective, and the  majority of immunohistochemistry analyses on uterine leiomyosarcoma are retrospective. Most specimens are also obtained following surgery. As was already said, having a precise diagnosis before to surgery is crucial for developing a surgical plan and improving prognosis. the cervical biopsy sampling technique used to validate the preoperative diagnosis. This aligns with the significant increase in scientific publications on alms after 2015, as depicted in **Figure 10**.

Combining with the Trend Topics (**Figure 6**), the research hotspots on uLMS in the past 3 years are roughly nomogram, case report, cell cycle, alk, and ultrasound etc., meanwhile, we can find that nomogram and cell cycle will still be the research hotspots of uLMS in 2023. The first two are related to the category of literature, while the latters, cell cycle, alk, and ultrasound, belong to basic research. Therefore, we believe that further discussion can be made about the research types of the current relevant literature on uLMS. Currently, the R package can only classify documents into categories such as articles, reviews, etc., and cannot perform deeper classification on articles. We used the search function in Excel to preliminarily classify the articles into case reports, clinical trials, nomogram, and basic research.

Case reports are a traditional genre of literature. There have been numerous case reports on leiomyosarcoma of the uterus in recent years. The vast majority of these case reports deal with unusual clinical characteristics of the tumor (66), uncommon distant metastases (67), treatment outcomes (68), etc. Upon reviewing these case reports, one discovers that the majority of them are case studies, making it impossible to categorize and condense uLMS. Nonetheless, the author discovered that throughout the last two decades, uLMS case reports had followed this criterion. The initial focus of specialists was on cases of solitary uLMS, but they also looked at cases of uLMS associated with other systemic disorders, like eosinophilia (69), dedifferentiated premenopausal leiomyosarcoma in ULMS following uterine artery embolization (70), and hematological diseases (71). The author suggest that specialists are focusing on these cases in part because uterine leiomyosarcoma cases are rare and difficult to diagnose. This is true even though the condition linked to these cases is not necessarily related to uterine leiomyosarcoma itself. Subsequently, it was gradually discovered that the majority of the case reports had something to do with metastasis, including cases of oral (72, 73), kidney (36), pancreatic (74), skin (75), and brain (76). All of the instances that involved long-term metastases fell into stage IV or higher of uLMS. Surgical resection is typically insufficient for this stage of uterine leiomyosarcoma, necessitating systemic therapy or external radiation control (3). In recent years, new worries concerning uLMS have surfaced, and clinical specialists have steadily concentrated on deeper levels, such as plag1-rearrangment-related cases (77), and the description of cell t features in rare metastatic cases (78). Examples include those linked to somatic BRCA2 mutations (79), smooth fibroids cases linked to ki-67, p53, and p16 (80), and cases where uterine leiomyomas treated with Ulipristal acetate led to the development of uLMS (81). A few novel uLMS treatment approaches were also mentioned in recent case reports. These included the use of eribulin mesylate to manage uLMS cases (82) and high-intensity focused ultrasound to treat recurrent uLMS (83). The author considers that the gradual pattern in uLMS case reports over the previous 20 years could be related to both the difficulties in identifying and treating uLMS and technological improvements. Stated differently, it may not always be simple to diagnose uLMS or uLMS conditions that are combined with other problems, nor will it always be simple to treat them. Scientists’ focus has gradually switched to improving prognosis as knowledge and technology have progressed. The public’s attention has been gradually drawn to the study of mechanisms and strategies for improving prognosis in recent years. However, the author points out that this also draws attention to a side issue, which is that there are still some holes in the pathogenesis research and difficulties with the diagnosis of uterine leiomyosarcoma (84). Genes that are increased in uLMS include matrix metalloproteinase 9 (MMP9), apolipoprotein E (apoE), cyclin E1, and conectin 1. Other research have examined the function of the SHARPIN gene, TP53, SLC39A7, GPR19, and other genes. There is a suggestion that they could be the cause of uLMS mutations (85). Nevertheless, to the best of our knowledge, no mechanistic studies pertaining to these chemicals or genes have been found. Numerous investigations have examined genetic alterations in LMS, such as those in TP53, MED12, and ATRX, and have found a correlation between these mutations and uLMS (86). The authors discovered, however, that the current investigation stuck to genome exon sequencing, validating RB1, TP53, and PTEN deletions and mutations from the TCGA database. It is worth highlighting that MED12 is a helpful biomarker for the diagnosis of uterogenic LMS and is relatively favorable for prognosis (84). These research are still at the level of big databases, and the author thinks that increasingly intricate experiments—like indepth investigations at the protein or mechanism pathway levels—are still required for verification.

With seven phase III clinical studies, phase II clinical trials make up the majority of clinical trials. The majority of these clinical trials concentrate on chemotherapy for uLMS. Phase II clinical trials have investigated the efficacy and safety of particular chemotherapy regimens, such as cisplatin, gemcitabine, docetaxel, and disrobing. Nevertheless, these trials’ statistically reliability is diminished by their small sample size. Therefore, further research into chemotherapeutic regimens for alms requires larger-scale phase III clinical studies. The topic trend also shows that substances like the cell cycle and alk have come to the forefront of basic research scientists’ attention. On uLMS, some researchers have discovered that aberrant activation of cell cycle-related kinases and inhibition of PLK1 or CHEK1 may have therapeutic benefits. Nevertheless, only mice have been used to validate this conclusion (87). Furthermore, researchers have identified ALK-fusion-containing uterine mesenchymal tumors and their reaction to ALK-targeted treatment (88). These results are consistent with the factorial analysis’s first cluster. Research on particular pathways for alms is still scarce, though. More thorough mechanism study will be helpful for uLMS diagnosis, treatment, and prognosis assessment (87). Nomograms are a relatively new population-based statistical prediction model that has seen a rise in use recently for prognosticating cancer. They reduce the statistical prediction model to a single numerical estimate, customized to the specific patient’s profile, of the chance of an event (such death or recurrence) (89). Nine articles on nomograms have been published in the last few years. The main reason they were created was because the existing AJCC and FIGO were not able to accurately predict cancer-specific survival (CSS) and overall survival (OS) in patients in a clinical context. Scientists used a nomogram derived from the Surveillance, Epidemiology, and End Results (SEER) database to address this. Age, ethnicity, tumor size, case tissue type, surgical staging, radiation status, and marital status were among the variables that this nomogram used in the model. Next, the model was applied to patients with uLMS in order to investigate and forecast OS (90) and CSS (91). This method is thought to be useful for predicting malignancies such as uLMS (90). The SEER database, which is used across this work, helps to offset the drawbacks of small sample sizes in basic and clinical research. It is crucial to remember that these research lack individual case reports and clinical trials and are mostly retrospective in nature. We may comprehend the state of research today, as well as potential future hotspots and trends, by having a thorough awareness of the many study kinds in the alms sector.

We acknowledge that our findings have several limitations that should be addressed in more extensive investigation. Firstly, we only employed Web of Science to search for publications in the previous 20 years and assessed reviews and articles entirely from Core+Zone 2 Sources, rejecting other search engines like Scopes, Google Scholar, or Index Medics. As a result, different sources could result in different quantities of research projects or citation counts. Second, the results of our study are only provisional and valid as of the data extraction date (January 6, 2024) because citation counts are prone to change over time. Thirdly, the percentage of cited papers should be taken into account when calculating the h-index and impact factor of authors, publications, journals, and nations. Fourthly, at this time, the further stages of analysis and classification of articles cannot be performed by the blistery package. Even though we partially addressed this issue in our study by using Excel’s filtering tool, a more accurate classification and thorough analysis are still necessary going forward. However, we are confident that our work provides a thorough investigation and advances knowledge of uLMS.

## Conclusion

In conclusion, bibliometrics stands out as an indispensable tool for delving into and comprehending the dynamic evolution of diverse scientific domains. Through its synergistic application of qualitative and quantitative analyses, augmented by sophisticated visualization techniques, bibliometrics facilitates a more profound exploration of research landscapes. This proves particularly invaluable in navigating the intricate terrain of uLMS and other scientific disciplines.

Our strategic choice of the Web of Science (WoS) as the primary database, owing to its expansive interdisciplinary coverage, centered on the past two decades of English literature. Leveraging “bibliometric version 4.1.0” and Citespace, our comprehensive analysis encapsulates 2489 pertinent articles, providing a comprehensive overview of the uLMS literature.

Highlighted by an annual publication average of 8.75, a commendable 6.07% yearly growth rate, and an average citation count of 17.22, our findings underscore the vibrant and evolving nature of uLMS research. The inclusion of Core+Zone 2 sources, contributing 1079 articles and 207 reviews with a 4.98% annual growth rate, further enriches our understanding of uLMS literature dynamics.

Our bibliometric analyses meticulously identified top journals, influential authors like *HENSLEY ML*, and core sources, emphasizing the prominence of GYNECOLOGIC ONCOLOGY and the substantial contributions from the United States. Bradford’s Law and Lotka’s Law offered insights into core sources and author productivity, respectively, augmenting the depth of our analysis.

The thematic map and factorial analysis, revealing distinct research clusters encompassing etiology, diagnosis, treatment advancements, and surgical approaches, contribute significantly to our nuanced understanding. Themes such as gemcitabine and docetaxel emerged prominently, adding valuable dimensions to the discussion.

In essence, this comprehensive bibliometric analysis not only unravels the nuanced trends within uLMS literature but also sheds light on influential factors shaping the field. The insights garnered herein lay a robust foundation for future research directions and contribute to the ongoing discourse within uLMS and related scientific domains.

## Data availability statement

The original contributions presented in the study are included in the article/**Supplementary Material**. Further inquiries can be directed to the corresponding author.

## Author contributions

JH: Formal analysis, Methodology, Software, Writing – original draft. YC: Conceptualization, Formal analysis, Funding acquisition, Methodology, Supervision, Writing – original draft, Writing – review & editing. XH: Investigation, Writing – review & editing, Visualization. ZL: Investigation, Visualization, Writing – review & editing. DH: Formal analysis, Software, Writing – original draft, Methodology. MC: Investigation, Visualization, Writing – original draft. YZ: Writing – original draft, Data curation. PZ: Writing – original draft, Data curation. XC: Project administration, Resources, Validation, Visualization, Writing – review & editing. ZY: Conceptualization, Funding acquisition, Writing – original draft, Supervision.
